# Transcriptomic analysis of mouse liver reveals a potential hepato-enteric pathogenic mechanism in acute *Toxoplasma gondii* infection

**DOI:** 10.1186/s13071-016-1716-x

**Published:** 2016-08-03

**Authors:** Jun-Jun He, Jun Ma, Hany M. Elsheikha, Hui-Qun Song, Si-Yang Huang, Xing-Quan Zhu

**Affiliations:** 1 State Key Laboratory of Veterinary Etiological Biology, Key Laboratory of Veterinary Parasitology of Gansu Province, Lanzhou Veterinary Research Institute, Chinese Academy of Agricultural Sciences, Lanzhou, Gansu Province 730046 People’s Republic of China; 2 Faculty of Medicine and Health Sciences, School of Veterinary Medicine and Science, University of Nottingham, Sutton Bonington Campus, Loughborough, LE12 5RD UK; 3 College of Veterinary Medicine, Hunan Agricultural University, Changsha, Hunan Province 410128 People’s Republic of China; 4 Jiangsu Co-innovation Center for Prevention and Control of Important Animal Infectious Diseases and Zoonoses, Yangzhou, Jiangsu Province 225009 People’s Republic of China

**Keywords:** *Toxoplasma gondii*, Mouse liver, Transcriptome, RNA-seq, Liver function

## Abstract

**Background:**

*Toxoplasma gondii* is a worldwide spread pathogen which can infect all tissues of its host. The transcriptomic responses of infected brain and spleen have been reported. However, our knowledge of the global transcriptomic change in infected liver is limited. Additionally, *T. gondii* infection represents a highly dynamic process involving complex biological responses of the host at many levels. Herein, we describe such processes at a global level by discovering gene expression changes in mouse livers after acute infection with *T. gondii* ToxoDB#9 strain.

**Results:**

Global transcriptomic analysis identified 2,758 differentially expressed transcripts in infected liver, of which 1,356 were significantly downregulated and 1,402 upregulated. GO and KEGG database analyses showed that host immune responses were upregulated, while the metabolic-related processes/pathways were downregulated, especially xenobiotic metabolism, fatty acid metabolism, energy metabolism, and bile biosynthesis and secretion. The metabolism of more than 800 chemical compounds including anti-*Toxoplasma* prescribed medicines were predicted to be modulated during acute *T. gondii* infection due to the downregulation of enzymes involved in xenobiotic metabolism.

**Conclusions:**

To the best of our knowledge, this is the first global transcriptomic analysis of mouse liver infected by *T. gondii.* The present data indicate that during the early stage of liver infection, *T. gondii* can induce changes in liver xenobiotic metabolism, upregulating inflammatory response and downregulating hepatocellular PPAR signaling pathway, altering host bile biosynthesis and secretion pathway; these changes could enhance host intestinal dysbacteriosis and thus contribute to the pathological changes of both liver and intestine of infected mice. These findings describe the biological changes in infected liver, providing a potential mechanistic pathway that links hepatic and intestinal pathologies to *T. gondii* infection.

**Electronic supplementary material:**

The online version of this article (doi:10.1186/s13071-016-1716-x) contains supplementary material, which is available to authorized users.

## Background

Toxoplasmosis, caused by the intracellular protozoan parasite *Toxoplasma gondii*, is a worldwide infectious disease that has the capacity to affect any warm-blooded vertebrate. To date, over 180 *T. gondii* genotypes have been identified. In North America and Europe, the widespread genotypes of *T. gondii* are the types I, II and III [1, 2]. ToxoDB#9, also known as genotype Chinese 1, is the predominant genotype in China [3, 4]. The wide host range, high prevalence and distinct clinical diversity of parasite genotypes represent a significant public health risk.

Although *T. gondii* exhibits striking neurotropic and ocular affinities, this parasite can also infect visceral organs, such as liver, spleen, pancreas, heart and lymph nodes. Hepatic toxoplasmosis does exist, but probably because *T. gondii* spreads to the liver during the acute phase of infection, this may go unnoticed. Indeed, several hepatic pathologies, such as hepatomegaly, hepatitis, granuloma, necrosis, cholestatic jaundice and cirrhosis have been linked to *T. gondii* infection [5–8]. *Toxoplasma gondii* infection has also been incriminated in causing abnormal liver function tests [9] and liver dysfunction in liver and kidney transplant recipients [10]. Acute *T. gondii* infection with RH strain in mice revealed an association between the increased number of hepatic stellate cells (HSCs) and the amount of *T. gondii* antigens, suggesting a regulatory role for HSCs in the pathogenesis of *T. gondii*-induced hepatitis [11].

Liver plays an important role in many biochemical and physiological processes, such as detoxification of xenobiotics or drugs and producing bile, which has a regulatory role in nutrition absorption, food digestion, maintaining gut bacterial balance and immune response [12]. On the other hand, some *T. gondii* strains have evolved to subvert host immune response [13]. For example, rhoptry protein 16 (ROP16) of Type I *T. gondii* can manipulate the production of host interleukin (IL)-12 and influence host IFN-γ production [14]. Thus, it is reasonable to hypothesize that *T. gondii* induces alteration in immune-regulatory and other genes in the hepatic tissues to evade host immune defense mechanisms during the early course of infection. Studying the global gene expression in liver of mice during acute *T. gondii* infection may uncover new facets of the immune-regulatory mechanisms that govern the interaction between *T. gondii* and the host.

Transcriptome analysis has been a powerful tool for the study of global biological changes in various body organs of mice infected with *T. gondii*. For example, Tanaka et al. applied RNA-seq to analyze global transcriptomic changes in mice brain infected with *T. gondii* [15]. Knight et al. used transcriptomics to elucidate the role of inflammation in *T. gondii*-inducing retinochoroiditis [16]. We previously analyzed the transcriptomic changes of infected mouse spleen to unravel the immune response to *T. gondii* infection [17]. However, despite the importance of the liver in the metabolism and other functions in the human body, the global transcriptomic changes of liver are still unknown. To better understand the molecular mechanisms that mediate the interaction between *T. gondii* infection and the liver, we used next-generation sequencing to assess the global transcriptomic gene expression in the liver of experimentally infected mice during the early course of infection. Our results revealed global gene expression changes, which are important in elucidating mechanisms linking liver and intestinal pathologies during early *T. gondii* infection. Full and complete understanding of these mechanisms will provide information likely to be critical for the development of rationally designed therapeutics or vaccines to mitigate this infection.

## Methods

### Animals, parasite challenge and sample collection

The PYS strain of *T. gondii* (genotype ToxoDB#9) is preserved and passaged in HFF cells in our laboratory. Six-week-old female BALB/c mice of special pathogen free (SPF) were purchased from Laboratory Animal Center of Lanzhou Veterinary Research Institute, Chinese Academy of Agriculture Science. Six mice were randomly divided into two groups (three replicates per group). Each mouse of the infected group was peritoneally infected with 200 tachyzoites of the PYS strain, while control mice were mock-injected with phosphate buffer saline (PBS). Mice were provided non-medicated feed and water *ad libitum* throughout the experiment. Six days post-infection (dpi) mice were humanely sacrificed by CO_2_ asphyxiation and liver tissues collected and stored at -80 °C until use. *Toxoplasma gondii* infection was confirmed by polymerase chain reaction (PCR) as described previously [18]. All mice were handled strictly in accordance with the Animal Ethics Procedures and Guidelines of the People’s Republic of China.

### Histopathological analysis and liver functional analysis

For histopathological analysis and liver functional analysis, twelve BALB/c mice were randomly divided into two groups (six replicates per group). Each mouse in the infected group was peritoneally infected with 200 tachyzoites of the PYS strain, while mice of the control group were mock-injected with PBS. All mice were humanely sacrificed by CO_2_ asphyxiation and the sera, livers were collected at six dpi. The collected livers were fixed in 10 % neutral buffered formalin solution for 1 week, paraffin-embedded and stained with haematoxylin and eosin. *Toxoplasma gondii* infection in liver was confirmed by PCR as described previously [18]. Serum albumin, globulin, adenosine deaminase activity, alkaline phosphatase activity, total bilirubin, total bile acid, high density lipoprotein, low density lipoprotein, cholinesterase activity, glutamic-pyruvic transaminase activity and glutamic-oxaloacetic transaminase activity were measured with the assistance of the No. 2 People’s Hospital of Lanzhou. Statistical analysis was performed by using SPSS Version 13.0 (SPSS Inc., Chicago, IL, USA). One sample Kolmogorov-Smirnov test was applied to test whether data exhibit normal distribution. Independent sample *t*-test was applied to analyze whether the variables mentioned above were significantly different between mice from infected versus control group. The data that did not follow a normal distribution were analyzed with Mann-Whitney test with Dunn’s multiple comparison *post-hoc* test; *P* < 0.05 was taken as significance cut-off.

### RNA extraction, sequencing and identification of differentially expressed transcripts (DETs)

Total RNA was prepared individually from the cryopreserved mice liver samples using TRIzol Reagent according to the manufacturer’s protocol (Invitrogen China Ltd, Beijing, China). All extracted RNA samples were treated with 20 units of RNase-Free DNase (Ambion, Shanghai, China) to remove residual genomic DNA according to the manufacturer’s recommendations. The integrity and quantity of all RNA samples were confirmed by Agilent 2100 Bioanalyzer (Agilent Technologies, Santa Clara, CA, USA) and Nanodrop 2000 (Thermo Scientific, Wilmington, DE, USA), respectively. Five micrograms RNA of each sample was individually used for the constructions of transcriptome libraries using IlluminaTruSeq™ RNA Sample Preparation Kit (Illumina, San Diego, CA, USA) and sequenced by using IlluminaHiSeq™2000 according to the manufacturer’s instructions. Q20 was used as quality control standard to filter raw reads. After filtering the low quality reads, the adaptors of high quality reads were removed and then clean reads were aligned to the mouse genome (mm10) using SOAP aligner/SOAP2 and GTF annotation data file (*Mus*_*musculus*.GRCm38.69). No more than five mismatches were allowed in the alignment. The gene expression level was calculated using RPKM method [19]. Differentially expressed transcript of each biological replicate was identified by using two-fold change (log2 fold-change ≥ 1 or ≤ -1), statistical test referring to the significance of digital gene expression profiles [20], and a 0.001 Benjamini & Hochberg False Discovery Rate (FDR) [21] corrected *P-*value cut-off for the thresholds. Only the transcript that was upregulated or downregulated in three biological replicates was identified as differentially expressed transcript (DETs). RNA isolation, library construction, RNA-sequencing (RNA-seq), reads alignment and DETs identification were performed at BGI-Shenzhen, China.

### Validation of RNA-seq results by quantitative real-time PCR (Q-PCR)

Gene expression data was further verified by Q-PCR. All six RNA templates that were used for RNA-seq were reverse transcribed to cDNA using M-MLV reverse transcriptase (Promega, Beijing, China) according to the manufacturer’s instructions, individually. β-actin was chosen as endogenous reference gene and ten genes were randomly selected for validation using Q-PCR. All Q-PCR reactions were performed on the Rotor-Gene Q (QIAGEN, Hilden, Germany) using SYBR Green GoTaq® qPCR Master Mix (Promega, Beijing, China) according to the manufacturer’s instructions. The selected genes were analyzed in triplicates and the primers used in this study are shown in Table 1. The Q-PCR cycle conditions were as follows: 95 °C for 5 min followed by 40 cycles of 95 °C for 10 s, 60 °C for 10 s, 72 °C for 20 s; melt curve analysis ranged from 72 °C to 95 °C to ensure that specific product was amplified in each reaction. The 2^−ΔΔCT^relative expression calculating method was used to calculate gene expression [22].

**Table 1:** Genes and primers used in Q-PCR validation

Gene	Accession No.	Primer name	Primer sequence (5′ to 3′)	Product length (bp)
Fbln5	NM_011812.4	Fbln5-F1	TAGAGCTCAAGGCTAGAAG	94
		Fbln5-R1	ACTTAGCGTCTCTACTCTG	
Car3	NM_007606.3	Car3-F1	CAGCCCTGGTCAGCATCT	145
		Car3-R1	ATTGGCGAAGTCGGTAGG	
Cyp2c69	NM_001104525.1	Cyp2c69-F1	CTGAGAAAGGCACGAAGT	232
		Cyp2c69-R1	GAATGAGGTCCAACGATA	
Atp6v0a4	NM_080467.3	Atp6v0a4-F1	ATTCTCAGCCTCTTCAATCA	195
		Atp6v0a4-R1	CAGAAACATGCCGATAAAGT	
Gbp2b	NM_010259.2	Gbp2b-F	AAATGGCCTCAGAAATCCAC	107
		Gbp2b-R1	TTGGATAGCAGACAGGATGT	
Maoa	NM_173740.3	Maoa-F1	AGATTTCTAAGCCTACCTGT	136
		Maoa-R1	GAGCCCTAATTTCATTCTGT	
Serpina6	NM_007618.3	Serpina6-F1	ACACCACCAAAGACACTCC	193
		Serpina6-R1	ATCATCAGGCTGCTCCAT	
Ifng	NM_008337.4	Ifng-F1	CCTGAAAGAAAGCAGTGTCT	85
		Ifng-R1	TTTGTCATTCGGGTGTAGTC	
Il1b	NM_008361.4	Il1b-F1	ACAGTGATGAGAATGACCTG	332
		Il1b-R1	GTAGTGCAGTTGTCTAATGG	
Cat	NM_009804.2	Cat-F1	GCAGTGATTTCACATAGGAT	131
		Cat-R1	GAGAGCTGGTAATCTCTACT	
β-actin	NM_007393.5	β-actin-F1	GCTTCTAGGCGGACTGTTAC	100
		β-actin-R1	CCATGCCAATGTTGTCTCTT	

Forward (F1) and reverse (R1) primers used for quantitative PCR

### Bioinformatics analysis of differentially expressed genes

Gene Ontology (GO) analysis of DETs was performed on the cytoscape plugin, BiNGO [23]. All data files used in BiNGO were downloaded from Gene Ontology database (http://www.geneontology.org/) [24]. Pathway analysis of DETs was performed through KEGG database (KEGG, http://www.genome.jp/kegg/) [25]. Hypergeometric tests and Benjamini & Hocherg False Discovery Rate (FDR) correction were performed to identify significantly enriched GO terms or KEGG pathways. The corrected *P* < 0.05 was used as a cut-off for the thresholds. Differently expressed GO terms or KEGG pathways were also identified where the FDR corrected *P-*value was less than 0.05, the number of DEPs was greater than 3, and the ratio between upregulated protein and downregulated protein was greater than 2 or less than 0.5. Comparative Toxicogenomics Database (http://ctdbase.org/) [26] was used for analysis of the relationship among xenobiotic metabolism, liver disease and DETs. Transcription factor analysis of DETs was carried out through Pscan web service (http://159.149.160.51/pscan/) [27]. In the Pscan web service, *Mus musculus* was selected in organism option and -950 + 50 region of transcriptional start site (TSS) was selected for transcription factor prediction.

## Results

### Confirmation of *T. gondii* infection in mice

All mice of the infected group exhibited signs indicative of toxoplasmosis, while mice in the control group remained normal, without any clinical changes. No mouse has died at 6 days post-infection. The results of PCR detection and DNA sequencing indicated that all livers of infected mice were positive for B1 gene of *T. gondii*, while those in the control group were PCR negative.

### RNA examination, transcriptomic features of mice liver and quantitative real-time PCR validation

**Fig. 1:**
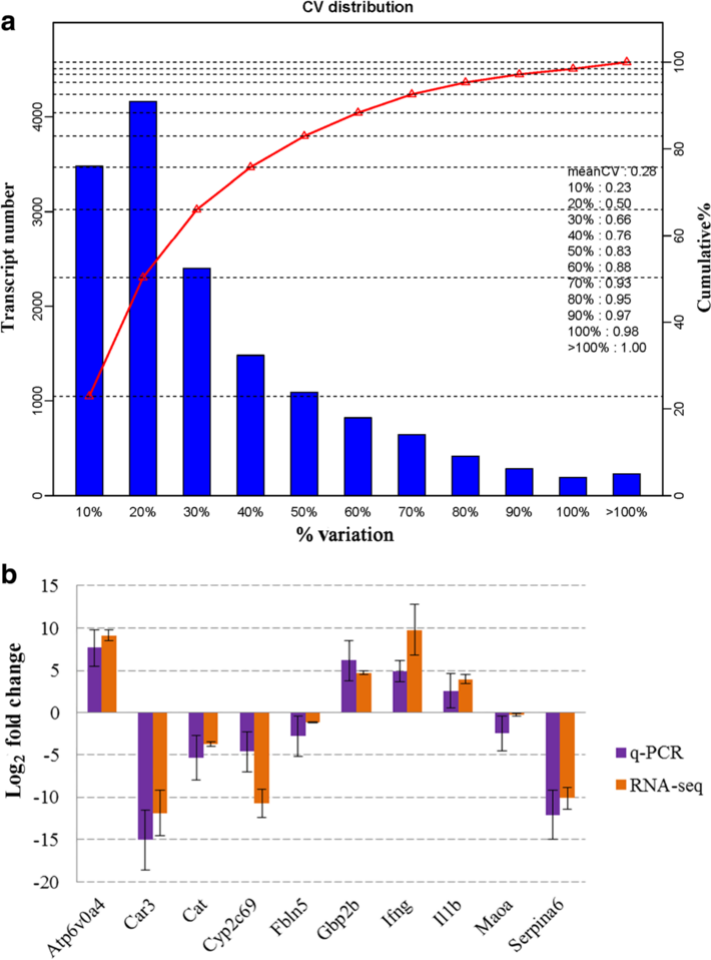
Coefficient of variation (CV) of the three replicates and validation of RNA-seq. **a** X-axis shows the % variation (*blue bars*) and *left Y-axis* shows the transcript number. *Right Y-axis* shows the cumulative variation (*red*). **b** Comparison of the results of ten transcripts between Q-PCR and RNA-seq. Bars indicate standard deviations

**Fig. 2:**
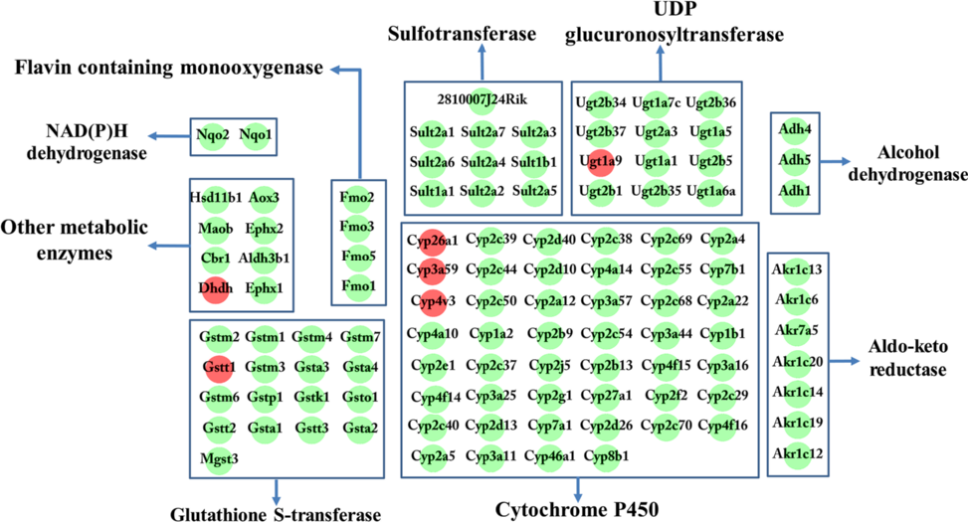
The differentially expressed genes involved in the metabolism of xenobiotics or drugs in the infected liver. The 109 differentially expressed genes involved in xenobiotic or drug metabolism were classed into nine groups, including UDP glucuronosyltransferases, sulfotransferases, glutathione S-transferases, cytochrome P450, flavin containing monooxygenases, aldo-ketoreductases, NAD(P)H dehydrogenases and other metabolic enzymes. *Green* spot represents downregulation, *red* spot represents upregulation

**Fig. 3:**
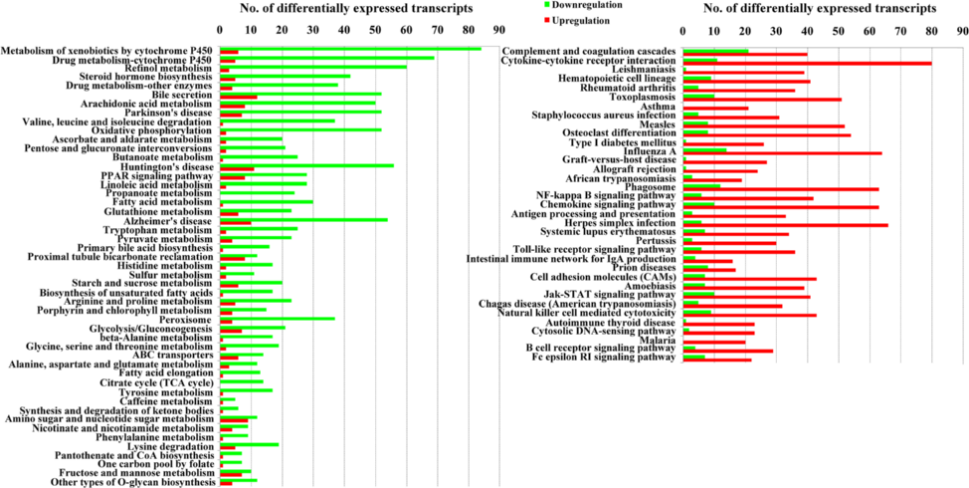
Significantly enriched pathways in *T. gondii*-infected liver. Eighty-four pathways were significantly enriched. *Green* represents downregulation, *red* represents upregulation

**Fig. 4:**
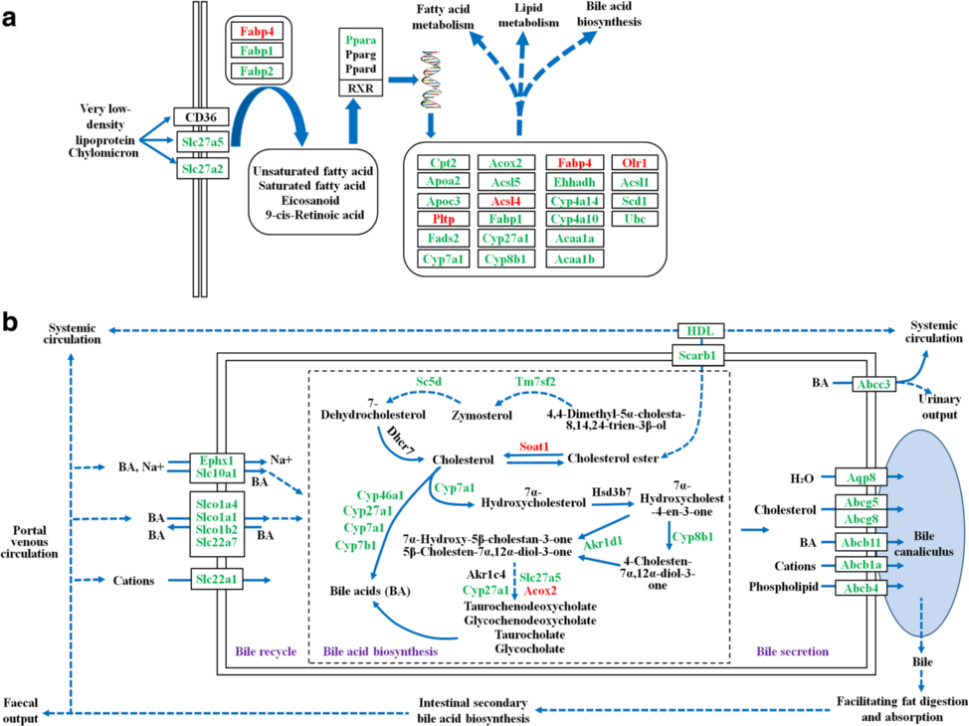
Downregulation of PPAR pathway and bile acid biosynthesis and secretion pathway. **a** Downregulation of PPAR signaling pathway. **b** Downregulation of bile acid biosynthesis and secretion pathway. *Solid arrows* indicate direct effect and *dotted arrows* indicate indirect effect. *Red* and *green* colour for genes indicate increased and decreased expression, respectively. The pathways were modified from the PPAR signaling pathway, the primary bile acid biosynthesis pathway and the bile secretion pathway of KEGG database. *Abbreviations*: BA, bile acid; HDL, high density lipoprotein

The RNA integrity number (RIN) of all six RNA templates used for RNA-seq was > 7. Over 47,000,000 clean reads were obtained in each mouse. The RNA-seq raw data are available at NCBI (accession no: PRJNA308347). More than 18,000 transcripts were identified. Coefficients of variation (CV) of the three biological replicates are shown in Fig. 1a. A total of 2,758 transcripts were identified as differentially expressed in the present study, including 1,356 downregulated transcripts and 1,402 upregulated transcripts. The details of the differentially expressed transcripts are shown in Additional file 1: Table S1. Results of ten randomly selected genes for Q-PCR validation are shown in Fig. 1b. No significant difference was found between results of the Q-PCR and RNA-seq. GO and KEGG enrichment analysis were applied to study the global biological change in infected liver. Our data indicate that 1,218 biological processes (Additional file 2: Table S2), 259 molecular functions (Additional file 3: Table S3), and 96 cellular components (Additional file 4: Table S4) were differentially expressed. Most upregulated GO terms were immune response related, while most downregulated GO terms were metabolic related, such as amino acid metabolic, xenobiotic metabolic, sterol metabolic, fatty acid metabolic, lipid metabolic and energy metabolic related GO terms. The differentially expressed enzymes involved in xenobiotic or drug metabolism are shown in Fig. 2. Consistent with GO enrichment analysis, all enriched pathways which involved in infection and immune response were upregulated, while the metabolism related pathways were downregulated in infected livers (Fig. 3). For example, both the upstreams (such as Slc27a2, Slc27a5, Fabp1, Fabp2 and Ppara) and downstreams (such as bile biosynthesis, fatty acid metabolism and lipid metabolism) of the PPAR signaling pathway were downregulated (Additional file 2: Table S2, Figs. 3 and 4a-b) in the present study.

### Comparative toxicogenomics database analysis of infected liver

In infected liver, over 800 chemical metabolism processes could be affected by the downregulated transcripts whose products are involved in xenobiotic or drug metabolism. The chemicals include narcotics, tranquilizers, ethanol, antibiotics, anti-inflammatory medicines, toxicants, lipids, acids, cancerogens and other chemicals. The relationship between the chemicals and differentially expressed genes that are involved in chemical metabolisms are listed in Additional file 5: Table S5. The Comparative Toxicogenomics Database analysis also gave us a list of liver-related diseases that may associate with *T. gondii* infection. In total, 39 liver-related diseases were shown in Toxicogenomics Database analysis, including carcinoma, cholestasis, liver cirrhosis, necrosis, drugs induced liver injury, fatty liver, fibrosis, inflammation and other diseases. The relationship between differentially expressed genes and liver diseases are listed in Additional file 6: Table S6.

### Liver inflammation, histopathological and functional analysis

**Fig. 5:**
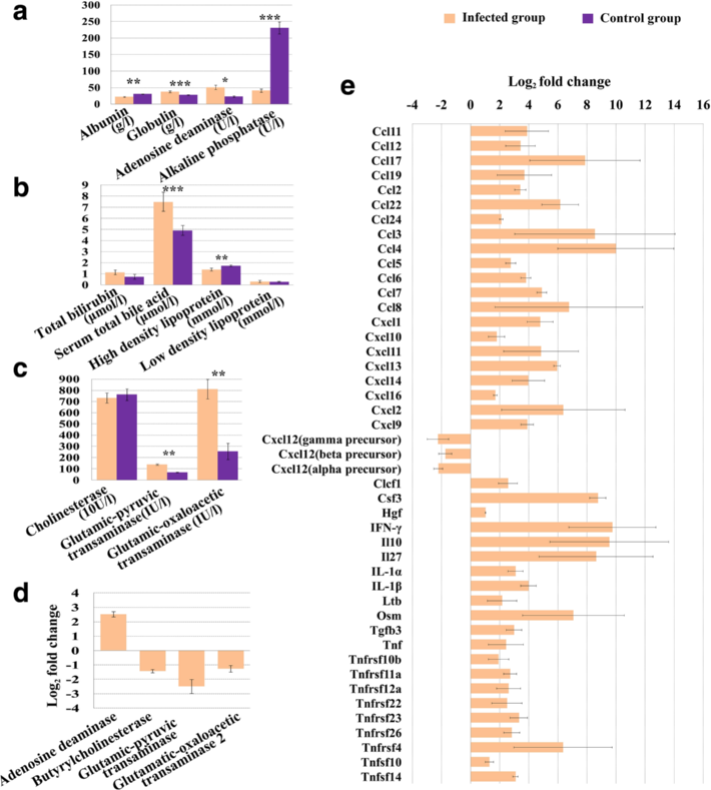
Results of liver functional analysis and the differentially expressed cytokines. **a**-**c** Levels of biochemical parameters in the mice serum. **d** The transcript change of adenosine deaminase (Ada), butyrylcholinesterase (Bche), glutamicpyruvic transaminase (Gpt), glutamatic-oxaloacetic transaminase 2 (Got2) in infected liver. **e** The differentially expressed cytokines in infected liver. Bars indicate standard deviations; units of biochemical parameters are shown along the X-axis. *Unit abbreviations*: g, gram; l, litre; U, unit; 10U, 10 units; UI, international unit; μmol, micromole; mmol, millimole. * Independent sample *t*-test, *P* < 0.05. ** Independent sample t-test, *P* < 0.01. *** Independent sample *t*-test, *P* < 0.001

**Fig. 6:**
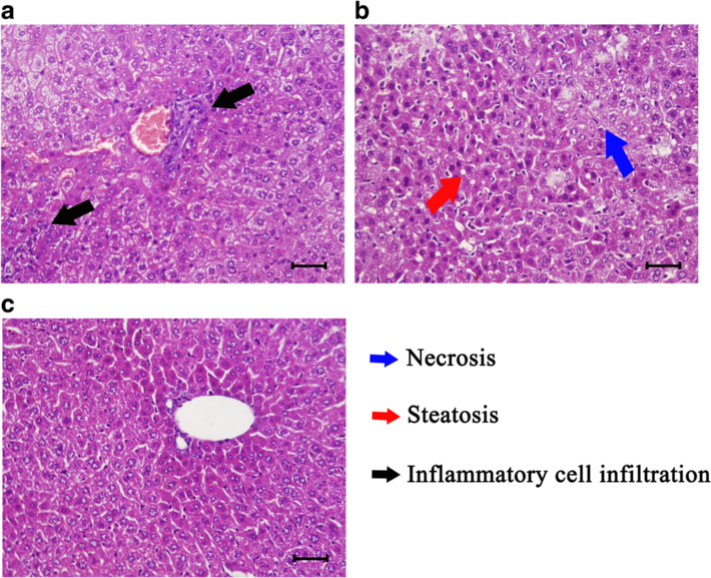
Pathological sections of mice liver. Histological section showing inflammatory cell infiltration (**a**), necrosis and slight steatosis in infected liver (**b**) compared to control mice (**c**). *Scale-bars*: 40 µm

The data of all liver functional test items followed a normal distribution. Globulin (Kolmogorov-Smirnov test *P* = 0.540; Independent sample *t*-test: *t*_(4)_ = 6.881, *P* < 0.0001), adenosine deaminase activity (Kolmogorov-Smirnov test *P* = 0.318; Independent sample *t*-test: *t*_(4)_ = 6.771, *P* = 0.015), total bile acid (Kolmogorov-Smirnov test *P* = 0.661; Independent sample *t*-test: *t*_(4)_ = 5.494, *P* < 0.0001), glutamic-pyruvic transaminase activity (Kolmogorov-Smirnov test *P* = 0.627; Independent sample *t*-test: *t*_(4)_ =17.748, *P* = 0.002) and glutamic-oxaloacetic transaminase activity (Kolmogorov-Smirnov test *P* = 0.81; Independent sample t-test: *t*_(4)_ = 8.383, *P* = 0.001) were significantly higher in infected mice serum, while albumin (Kolmogorov-Smirnov test *P* = 0.24; Independent sample *t*-test: *t*_(4)_ = -9.280, *P* = 0.009), alkaline phosphatase activity (Kolmogorov-Smirnov test *P* = 0.633; Independent sample *t*-test: *t*_(4)_ = -17.501, *P* < 0.0001) and high density lipoprotein (Kolmogorov-Smirnov test *P* = 0.751; Independent sample *t*-test: *t*_(4)_ = -4.988, *P* = 0.002) were significantly lower in the infected group (Fig. 5a-d). As shown in Fig. 6, mouse liver was damaged by *T. gondii* infection. Inflammatory cell infiltration, hepatocellular necrosis and liver steatosis were observed in mice of the infected group (Fig. 6a, b). There were 43 cytokines differentially expressed in infected liver, most of these upregulated (Fig. 5e). No histopathological change was observed in control liver (Fig. 6c).

### Transcription factor analysis of infected liver

**Fig. 7:**
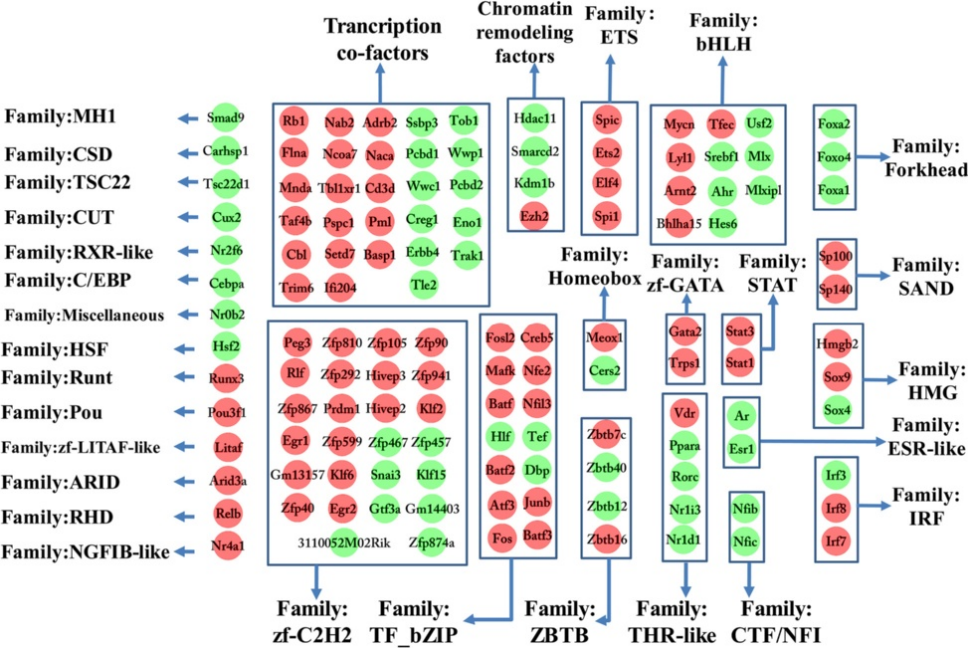
The differentially expressed transcription factors in infected liver. 131 transcription factors were differentially expressed and classified into 31 families. *Green* spot represents decreased expression and *red* spot indicates increased expression

In the present study, 99 transcription factors, four chromatin remodeling factors and 28 transcription co-factors were differentially expressed in infected livers; these can be clustered into 31 subsets according to the their family (Fig. 7). In Pscan analysis, the top ten transcription factors used by upregulated genes were Spi1, Erg, Ets1, Ehf, Nfkb1, Stat2, Stat1, Klf1, Fli1 and Irf1, while the top ten transcription factors used by downregulated genes were Hnf4a, Hnf4g, Sp2, Sp1, Klf5, Nr2f1, Klf4, Nr2c2, Pparg and Rxra.

## Discussion

We used next-generation sequencing to analyze the global transcriptomic change in the liver of mice infected with *T. gondii*. A total of 2,758 transcripts were differentially expressed in infected liver and our q-PCR validation confirmed the differentially expressed (Fig. 1b). Infected livers showed differential gene expression in pathways classified in the immune response (upregulated) and xenobiotic metabolism, fatty acid metabolism, energy metabolism, and bile biosynthesis and secretion response (downregulated).

Inflammation is the backbone of immune response which eliminates *T. gondii* infection via stimulating various immune cells to secret various cytokines and chemokines. Even though we observed inflammatory cells in pathological sections, identification of the different cell types in haematoxylin & eosin-stained sections was not possible. However, according to a published report [28], it is apparent that many cells including Th1, Th17, Treg cells, neutrophils, monocytes, macrophages, basophils, immature dendritic cells (iDCs), dendritic cells (DCs) and natural killer cells (NKs) could infiltrate to infected liver for the upregulation of chemokines (such as CXCL9, CXCL10, CXCL11, CXCL13, CXCL16, CXCL14, CCL19, CCL2, CCL3, CCL4 and CCL5). Overall, 43 cytokines were differentially expressed in infected liver, most of them were upregulated, including IFN-γ, IL-1β (Fig. 5e). DCs produce IL-12 after encountering *T. gondii* and stimulate NK cells to produce IFN-γ as well as promote the development of Th1 cells, which are important in adaptive immunity. However, over production of IL-12 and IFN-γ can cause lethal tissue damage. Th1 cells can produce IFN-γ through activation of myeloid differentiation primary-response protein 88 (Myd88) pathway and neutrophils produce IFN-γ in IL-1β-depend manner [29, 30]. Although IL-1β was upregulated in present study, the extent to which neutrophil contributes to IFN-γ production in infected liver is still unknown. Il1rn, which functions as interleukin 1 receptor antagonist and blocks IL-1β signaling transduced into neutrophil [31], was also upregulated. The upregulation of Il1rn was also observed in infected mouse spleen [17].

IFN-γ is the most important cytokine that eliminates intracellular *T. gondii* by upregulating three pathways: (i) the immunoregulatory enzyme indoleamine 2,3-dioxygenase (IDO) to deplete local cellular tryptophan; (ii) inducible nitric oxide synthase (iNOS) to limit cellular arginine; (iii) interferon inducible immunity related GTPases (IRGs) and guanylate binding proteins (GBPs) to destroy the parasitophorous vacuole membrane (PVM), which is used by *T. gondii* to acquire nutrients, hijack host organelles and protect the parasite from host immune systems [29, 32]. Notably, our study revealed that IFN-γ (Ifng), two iNOS (Nos2 and Nos3), one IDO (Ido1), seven IRGs (Irgm1, Irgm2, Iigp1, 9930111J21Rik2, Tgtp1, Tgtp2 and Gm12250) and nine GBPs (Gbp2, Gbp2b, Gbp3, Gbp4, Gbp5, Gbp6, Gbp8, Gbp9 and Gbp10) were upregulated. For innate immune pathways, toll-like receptor signaling pathway, NK cell mediated cytotoxicity and cytosolic DNA-sensing pathway were also upregulated (Fig. 3). The importance of toll-like receptor signaling pathway and NK cells for anti-*T. gondii* infection have been discussed in a previous review [29]. Further research is needed to elucidate the involvement of the cytosolic DNA-sensing pathway in *T. gondii* infection.

C-type lectin signaling pathway is another pathway that links innate immune response and adaptive immune response. Two recent reports showed that C-type lectin can restrict *T. gondii* parasitism [33, 34]. In the present study, ten C-type lectin molecules were differentially expressed, including eight upregulated C-type lectins (Clec12a, Clec1b, Clec4a1, Clec4a2, Clec4a3, Clec4e, Clec4n and Clec7a) and two downregulated C-type lectins (Clec11a and Clec3b). The function of Clec7a which contributes to *T. gondii* resistance has been discussed previously [34]. However, the functions of other differentially expressed C-type lectins are still unclear. Further studies should enhance our understanding of the role of C-type lectins in *T. gondii* infection.

Inflammation helps the host to eliminate *T. gondii* infection, at the same time, it can cause liver damage and impact bile biosynthesis [35]. Our histopathological analysis, liver functional test and transcriptomic analysis confirmed the liver inflammation and damage associated with *T. gondii* infection. For example, infiltrating inflammatory cell (Fig. 6a), necrosis of hepatocyte and liver steatosis (Fig. 6b) were observed in infected liver. Additionally, serum bile acid, adenosine deaminase and transaminase activity were upregulated in infected mice (Fig. 5a-c). The upregulation of serum bile acid, adenosine deaminase and transaminase activity is a sensitive biomarker of hepatocellular injury [36, 37]. Previous report confirmed that the upregulated serum bile acid results from the inability of hepatocyte to remove bile acid from serum [38]. As shown in Fig. 4b, hepatocellular bile reabsorption process was reduced by the downregulation of bile transporters, such as Ephx1, Slc10a1, Slco1a4, Slco1a1, Slco1b2, Slc22a7 and Slc22a1. In addition, hepatocellular bile acid biosynthesis and secretion process was also downregulated by the downregulation of Tm7sf2, Sc5d, Cyp46a1, Cyp27a1, Cyp7a1, Cyp7b1, Cyp8b1, Akr1d1, Slc27a5, Scarb1, serum high density lipoprotein (HDL), Abcc3, Aqp8, Abcg5, Abcg8, Abcb11, Abcb1a and Abcb4.

**Fig. 8:**
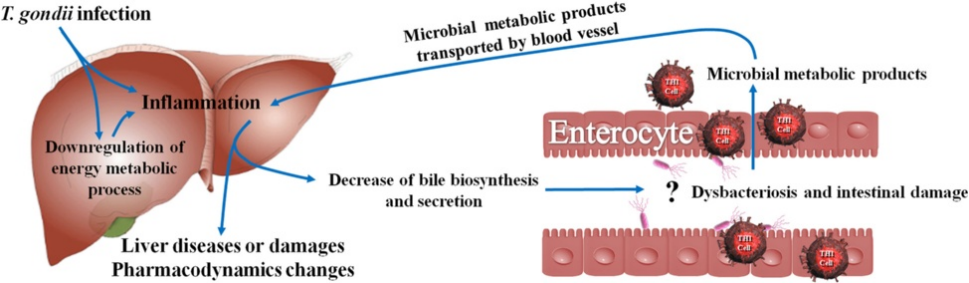
Potential link between *T. gondii* infection and liver injury and intestinal pathology. Question mark indicates a potential link

Downregulation of bile acid biosynthesis and secretion could result in low level of bile entering host intestine, which favors gram-negative bacteria and induces intestinal dysbacteriosis and pathology [39]. This finding is consistent with the fact that *T. gondii* infection increases intestinal gram-negative bacteria population [40]. Several reports have confirmed that *T. gondii* infeciton can induce mice intestinal pathological changes [40–45] and the increasing intestinal gram-negative bacterial population is one of the intestinal pathological inducers [40]. Biological processes in both liver and intestine are closely connected. The presence of intestinal pathology allows intestinal bacterial metabolic products to leak via blood vessel to liver and function as a positive feedback to enhance liver inflammation [46]. Our results suggest that *T. gondii* infection in liver can influence host health via inducing hepatic inflammation, decreasing bile biosynthesis and secretion, and ultimately could contribute to intestinal dysbacteriosis described previously and intestinal damage [40] which forms a cycle of hepatic inflammation and intestinal pathology (Fig. 8).

Bile biosynthesis and secretion is also associated with serum alkaline phosphatase activity and the bile acid is one inducer of alkaline phosphatase [47]. In the present study, serum alkaline phosphatase activity was downregulated. So the downregulation of bile acid biosynthesis and secretion could contribute to the low serum alkaline phosphatase activity in infected mice.

In addition to bile biosynthesis and secretion, another important function of the liver is drug or xenobiotic degradation. Generally, there are three phases for drug or xenobiotic metabolism in the liver. In phase I lipophilic molecules are transformed into hydrophilic molecules mediated by cytochrome P450 and NADPH. The metabolites of phase I are often toxic and carcinogenic. These must be transformed into nontoxic material via phase II, which is mediated by transferases and oxidoreductases, such as UDP glucuronyltransferases, glutathione-S-transferases, sulfotransferases, alcohol dehydrogenases, aldo-ketoreductases, and flavin-containing monooxygenases. In the phase III, all final products of phase II reactions are excreted into bile by export pumps. Inflammation in the liver can contribute to decreased drug metabolism [48]. Downregulation of macrolide antibiotics metabolisms and cyclosporin A metabolism has been observed in *T. gondii* infection [49, 50]. However, it is unclear how many enzymes contribute to the downregulation. Our study will fill the gaps.

In the present study, most differentially expressed genes involved in xenobiotic or drug metabolisms, such as cytochrome P450, NAD(P)H dehydrogenases, glucuronyltransferases, glutathione-S-transferases, sulfotransferases, alcohol dehydrogenases, aldo-ketoreductases, flavin-containing monooxygenases, and other genes involved in xenobiotics or drug metabolic process, were downregulated (Fig. 2). Understanding the xenobiotics or drug metabolic kinetics for patients is very important, because their alteration in liver may influence the response of treated individuals to the prescribed medicines, e.g. overdosed acetaminophen usually induces acute liver failure [51]. The results of Comparative Toxicogenomics Database analysis showed that 71 out of 73 differentially expressed enzymes involved in acetaminophen metabolism were downregulated in the infected liver. This indicates that the problem of acetaminophen inducing acute liver failure could be exacerbated in toxoplasmosis patients due to the pharmacokinetic changes. In addition, overdose of acetaminophen can induce mitochondrial DNA damage [52] or permeability transition which could induce liver diseases [53].

Therefore, in order to avoid adverse drug reaction in toxoplasmosis patients, the balance between medicinal effect and toxic effect of the drug should be considered in the light of any expected pharmacokinetic changes. According to Comparative Toxicogenomics Database analysis, a list of drugs or chemicals whose metabolism could be affected in *T. gondii* infection is provided. The details of the relationship between substrates and metabolic enzyme are listed in Additional file 5: Table S5. Although we did not directly test the pharmacokinetics changes in infected mouse, mRNA can reflect the change of its product [54], and hopefully our analysis could provide valuable data for helping toxoplasmosis patients to choose medicine and to avoid potential adverse drug reactions.

Gene transcription is regulated by cellular transcription factors. Studying transcription factor usage could be helpful for uncovering the relationship between *T. gondii* infection and liver functional changes. In the present study, 99 transcription factors, four chromatin remodeling factors and 28 transcription co-factors were differentially expressed. Some families of transcription factors showed common changes. For example, Forkhead, CTF/NFI and ESR-like families were downregulated while ETS, SAND, STAT and zf-GATA families were upregulated (Fig. 7). Through comparative analysis of the top ten usages of transcription factors between upregulated and downregulated genes, we detected a bias character of transcription factor usage. Top ten transcription factors used in upregulated gene expressions were Spi1, Erg, Ets1, Ehf, Nfkb1, Stat2, Stat1, Klf1, Fli1 and Irf1, while top ten transcription factors involved in downregulated gene expressions were Hnf4a, Hnf4g, Sp2, Sp1, Klf5, Nr2f1, Klf4, Nr2c2, Pparg and Rxra. According to KEGG database analysis, Pparg and Rxra are the components of PPAR signaling pathway, which plays important roles in regulating host bile biosynthesis, fatty acid metabolism, lipid metabolism and energy metabolism. In our study, both the upstreams and downstreams of the PPAR signaling pathway were downregulated. Fatty acid and lipid metabolism are both important sources of energy. Disruption of PPAR pathway can impair host energy production [55–57]. In our study, the energy related GO terms or pathways, such as hepatocellular ATP biosynthetic process, tricarboxylic acid cycle, fatty acid metabolism, lipid metabolism, pyruvate biosynthetic process, proton-transporting ATP synthase complex, mitochondrial respiratory chain complex I and III, CoA dehydrogenase or carboxylase activity and oxidative phosphorylation pathway, were downregulated (Additional files 2, 3 and 4: Tables S2, S3 and S4; Fig. 3). A severe impairment of energy production in hepatocytes could result in a series of injuries, such as liver necrosis, fatty liver and inflammation [53]. These pathological changes are consistent with the results of Comparative Toxicogenomics Database analysis (Additional file 6: Table S6) and our histopathological analysis (Fig. 6a, b). The PPAR pathway also participates in host anti-inflammation process [58]. Our results of transcription factor analysis showed that *T. gondii* could contribute to host liver systemic changes via downregulated host hepatocellular PPAR signaling pathway.

## Conclusions

Our study identified 2,758 differentially expressed transcripts in infected liver, including 1,356 downregulated transcripts and 1,402 upregulated transcripts. Infected liver showed upregulation of immune-related GO terms, immune response pathways and downregulation of metabolic-related GO terms and pathways. Pharmacokinetics of more than 800 chemical (including some prescription drugs) could be altered due to the downregulation of enzymes involved in the xenobiotic metabolism. Our findings also demonstrated downregulation of hepatocellular fatty acid metabolism, lipid metabolism, energy metabolism, bile biosynthesis and secretion. The downregulation of bile biosynthesis and secretion suggests a potential mechanistic pathway in mice linking liver and intestinal pathologies during acute *T. gondii* infection. Comparative analysis of transcription factor usage between upregulated and downregulated genes suggests that *T. gondii* could hijack hepatocellular PPAR signaling pathway to alter host liver biochemical processes. Although the response of mice liver may not be specific to *T. gondii* infection, our results provide valuable data for studying the response in the infected mouse liver.

## Abbreviations

CV, coefficients of variation; DETs, differentially expressed transcripts; dpi, days post-infection; FDR, false discovery rate; GO, gene ontology database; HSCs, hepatic stellate cells; PBS, phosphate buffer saline; PCR, polymerase chain reaction; Q-PCR, quantitative real-time PCR; RIN, RNA integrity number; RNA-seq, RNA sequencing; SPF, special pathogen free; TSS, transcriptional start site

## Additional files

Additional file 1: Table S1. Differentially expressed transcripts. (XLSX 688 kb) Additional file 2: Table S2. Differentially expressed biological processes. (XLSX 188 kb) Additional file 3: Table S3. Differentially expressed molecular functions. (XLSX 37 kb) Additional file 4: Table S4. Differentially expressed cellular components. (XLSX 25 kb) Additional file 5: Table S5. Interaction between chemicals and differentially expressed genes involved in xenobiotics or drugs metabolism. (XLSX 188 kb) Additional file 6: Table S6. Relationship between differentially expressed genes and liver diseases. (XLSX 44 kb)
